# Flipping the switch: tools for detecting small molecule inhibitors of staphylococcal virulence

**DOI:** 10.3389/fmicb.2014.00706

**Published:** 2014-12-12

**Authors:** Cassandra L. Quave, Alexander R. Horswill

**Affiliations:** ^1^Department of Dermatology, Emory University School of MedicineAtlanta, GA, USA; ^2^Center for the Study of Human Health, Emory University College of Arts and SciencesAtlanta, GA, USA; ^3^Department of Microbiology, Roy J. and Lucille A. Carver College of Medicine, University of IowaIowa City, IA, USA

**Keywords:** accessory gene regulator, *Staphylococcus aureus*, auto inducing peptides, drug discovery, toxins, quorum sensing, adjuvant therapy, quorum sensing inhibitor

## Abstract

Through the expression of the accessory gene regulator quorum sensing cascade, *Staphylococcus aureus* is able to produce an extensive array of enzymes, hemolysins and immunomodulators essential to its ability to spread through the host tissues and cause disease. Many have argued for the discovery and development of quorum sensing inhibitors (QSIs) to augment existing antibiotics as adjuvant therapies. Here, we discuss the state-of-the-art tools that can be used to conduct screens for the identification of such QSIs. Examples include fluorescent reporters, MS-detection of autoinducing peptide production, agar plate methods for detection of hemolysins and lipase, High performance liquid chromatography-detection of hemolysins from supernatants, and cell-toxicity assays for detecting damage (or relief thereof) against human keratinocyte cells. In addition to providing a description of these various approaches, we also discuss their amenability to low-, medium-, and high-throughput screening efforts for the identification of novel QSIs.

## INTRODUCTION

*Staphylococcus aureus* is an opportunistic pathogen that is the causative agent of numerous acute and chronic infections (Skov et al., 2012; Uhlemann et al., 2014). The prevalence of these infections has increased due to higher rates of colonization, immunosuppressive conditions, greater use of surgical implants, and dramatic increases in antibiotic resistance. More recently, methicillin resistant *Staphylococcus aureus* (MRSA) strains expanded from healthcare settings and began infecting otherwise healthy individuals in the community. These strains were coined “community-associated” MRSA (CA-MRSA) for their new properties and have become the most recent epidemic wave of resistance in *S. aureus* (Chambers and Deleo, 2009; DeLeo and Chambers, 2009). Outbreaks of CA-MRSA have spread worldwide with remarkable speed and have affected otherwise healthy individuals (Hidron et al., 2009; Yamamoto et al., 2010). Indeed, CA-MRSA infections confer a substantial clinical and economic burden, with total costs in the United States (US) estimated at over $15 billion US dollars per year (Lee et al., 2013). Given our knowledge of how quickly drug resistance spreads in *S. aureus*, it is apparent that we are rapidly exhausting current treatment options.

**FIGURE 1:**
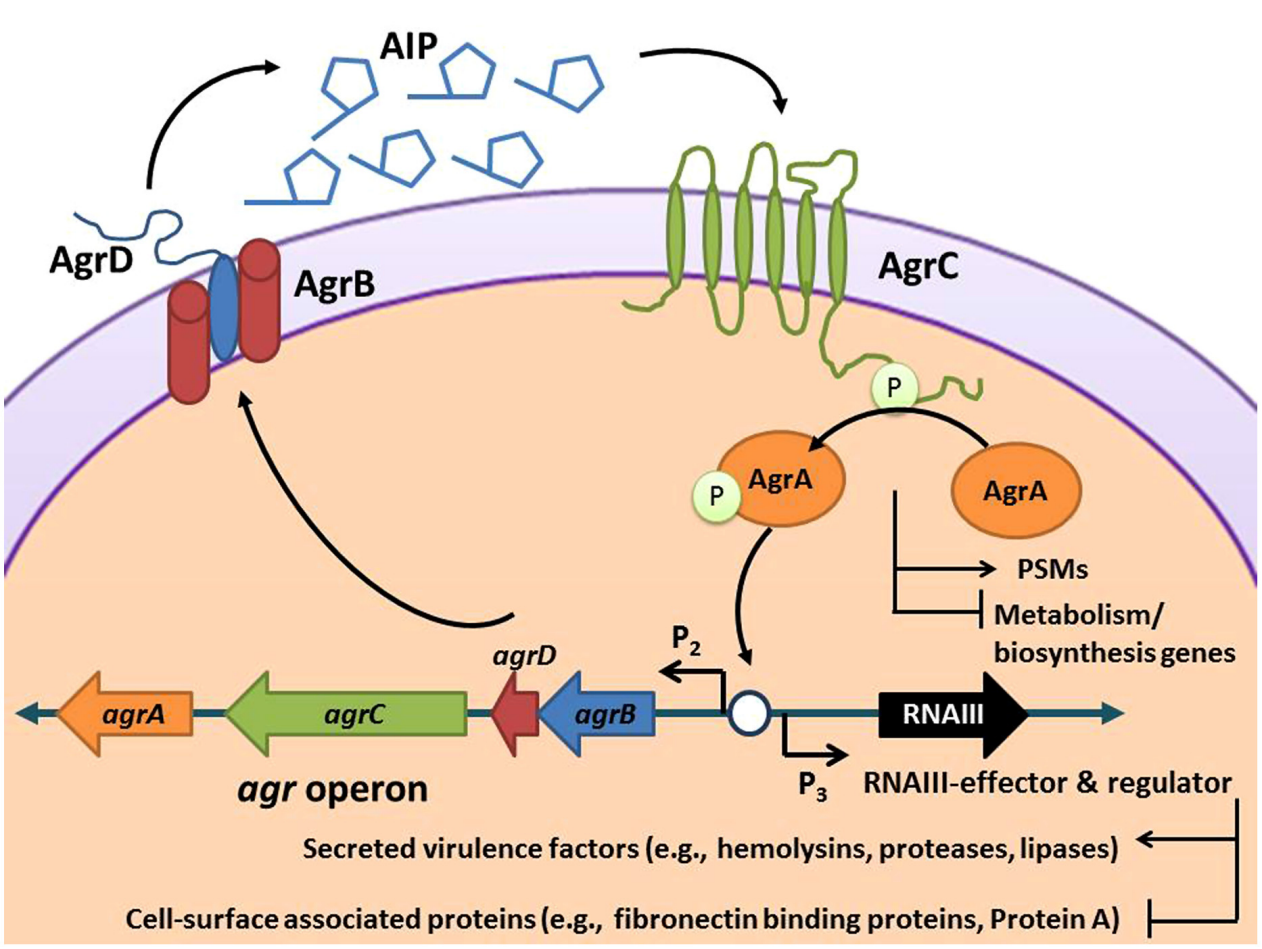
**Schematic of the *Staphylococcus aureus* accessory gene regulatory (*agr*) system.** The *agr* locus is known to contain two divergent transcripts named RNAII and RNAIII. The RNAII transcript is an operon of four genes, *agrBDCA*, that encode factors required to synthesize AIP and activate the regulatory cascade. Briefly, AgrD is the precursor peptide of AIP, AgrB is a membrane protease involved in generating AIP, AgrC is a histidine kinase that is activated by binding AIP, and AgrA is a response regulator that induces transcription of RNAII and RNAIII through the P2 and P3 promoters, respectively. AgrA also directly promotes PSM production. The RNAIII transcript yields a regulatory RNA molecule that acts as the primary effector of the *agr* system by up-regulating extracellular virulence factors and down-regulating cell surface proteins (Novick et al., 1993).

The hypervirulent nature of CA-MRSA is due to its active cell–cell communication pathway, or quorum sensing system, which controls expression of an extensive array of enzymes, hemolysins, and immunomodulators that are essential to its ability to spread through tissues and cause disease (Novick, 2003). These virulence factors serve a wide scope of purposes in the infection process, including disruption of the epithelial barrier, inhibition of opsonization by antibody and complement, neutrophil cytolysis, interference with neutrophil chemotaxis, and inactivation of antimicrobial peptides (Tsuji et al., 2007; Spaan et al., 2013; Foster et al., 2014; Otto, 2014). The expression of all of these invasive factors is controlled by cell-density quorum sensing using the autoinducing peptide (AIP) molecule. Like other quorum sensing signals, AIP accumulates outside the cell until it reaches a critical concentration and then binds to a surface receptor called AgrC, initiating a regulatory cascade (**Figure 1**). Since AIP controls the expression of accessory factors for *S. aureus,* this regulatory system has been named the accessory gene regulator (*agr*), and the majority of the proteins necessary for this quorum sensing system to function are encoded in the *agr* chromosomal locus (Novick, 2003; Thoendel et al., 2010). The use of small molecule inhibitors to “flip the *agr* switch” off and quench this communication system to attenuate pathogenicity and virulence lies at the core of the anti-virulence approach (Zhu and Kaufmann, 2013).

Despite recognition of the important role of *agr* regulation in *S. aureus* pathogenesis, to date, no quorum sensing inhibitor (QSI) candidates have made it to the clinic (Zhu and Kaufmann, 2013). However, efforts dedicated to the discovery of small molecule inhibitors of this system are currently underway in many labs, and have already resulted in the discovery of several promising leads (**Table 1**). These QSIs were identified through screens of synthetic compounds and natural products of various origins (i.e., fungal, botanical, microbial, and marine sources), see for example: (Quave et al., 2011; Murray et al., 2014; Nielsen et al., 2014; Sully et al., 2014). In this article, we aim to review the various tools being used in these ongoing efforts to identify novel inhibitors of the *S. aureus agr* system.

**Table 1:** Examples of reported inhibitors of the *Staphylococcus aureus agr* system.

Process or Agent	Mechanism	Reference
AIP analogs (many types)	AgrC antagonists	Reviewed in Gordon et al. (2013)
cyclo(L-Tyr-L-Pro) and cyclo(L-Phe-L-Pro)	Unknown; perhaps AgrC antagonist	Li et al. (2011)
Solonamide A and B	AgrC antagonists	Nielsen et al. (2014)
Ambuic acid	AgrB inhibitor	Nakayama et al. (2009)
Savirin	AgrA inhibitor	Sully et al. (2014)
Polyhydroxyanthraquinones	Unknown	Figueroa et al. (2014)

## STRAINS AND MUTANT CONTROLS

One of the most important aspects of investigating the *agr* system is the use of appropriate strains and controls. The USA300 (*agr* Type I) strains have a very robust *agr* system and produce consistently high levels of RNAIII (Li et al., 2009). Considering these strains are clinically relevant, the USA300s are excellent testing and screening strains for QSIs due to the large dynamic range of quorum sensing function. For confirmation, complete deletions of the *agr* system are available in USA300 (Lauderdale et al., 2009, 2010; Pang et al., 2010), and these mutants can be used to assess the *agr* selectivity of an inhibiting agent, as was done recently with the compound savirin (Sully et al., 2014). In testing the therapeutic efficacy of a QSI, the *agr* mutants are also important controls in animal models of infection to determine the importance of quorum sensing during host interactions (Thoendel et al., 2010). As a small-molecule control, the competing AIP-II or AIP-III signal serves as a low nanomolar inhibitor of the AgrC receptor, and these can be easily synthesized for studies (Mayville et al., 1999). For other *agr* Type I strains, older isolates like NCTC8325-4 and Newman have been used in many pioneering studies on *agr* function (Thoendel et al., 2010). While there have been tremendous advances made in these strains, they do have some limitations, such as the *rsbU* deletion in 8325-4 that greatly enhances RNAIII output (Lauderdale et al., 2009), potentially skewing the interpretation of inhibitor potency. More recently, there has been effort to fix these issues by repairing chromosomal mutations, with a goal of having a wild-type strain amenable to laboratory research (Herbert et al., 2010).

Going beyond the *agr* Type I strains, the availability of useful isolates, tools, and knowledge greatly diminishes. Although *agr* Type II strains were discovered early (Ji et al., 1997), they have received sparingly little attention in ongoing studies. The 1963 isolate 502A (often called SA502A or SA502a) has been the ‘go to’ strain for essentially all *agr* Type II studies. This strain is genetically tractable and *agr* reporters are available (Kirchdoerfer et al., 2011), and more recently the genome has been sequenced (Parker et al., 2014). However, the focus on 502A is somewhat misplaced considering recent clinical USA100 isolates are also *agr* Type II and represent over 53% of many hospital MRSA collections (Limbago et al., 2009). Similarly, for *agr* type III strains, USA400 MW2 has become the strain of choice for studies. It is a relevant clinical isolate (Baba et al., 2002), has a robust *agr* system, and *agr* mutants and reporters are available (Kirchdoerfer et al., 2011; Olson et al., 2013), making it an excellent strain for ongoing investigation and inhibitor testing. The *agr* type IV system is the rarest in terms of global distribution of *S. aureus* isolates (Holtfreter et al., 2007). Strains with reporters are available (Kirchdoerfer et al., 2011), but few other tools or mutants have been developed. When using any of these strains, caution is suggested due to the tendency of the *agr* system to mutate (Shopsin et al., 2008). Frequent testing of hemolytic activity and reconstruction of reporters is often necessary in order to draw accurate conclusions about a QSI candidate.

## THE QSI DETECTOR TOOLBOX

Following a review of the literature on the staphylococcal *agr* system, we have identified four major groups of *in vitro* and *ex vivo* tools used for assessing *agr* function: agar plate assays, cell culture and *ex vivo* techniques, chromatographic tools and molecular tools. These assays differ in cost, sensitivity, and amenability to screening of small molecules for QSI activity (**Table 2**). For the purposes of clarity, here we delineate the characteristics considered during our categorization of techniques as being low-, medium-, or high- throughput:

**Table 2:** Summary of existing tools for assessing *agr* function.

Assay	Throughput^a^			EE^b^	SM^c^	Reference^d^
	L	M	H			
**Agar Plate Assays**
CAMP test	✓					Elek and Levy (1950)
β-hemolytic disk assay for δ-hemolysin	✓					Schweizer et al. (2011)
Phospholipase plate assay	✓					Kouker and Jaeger (1987); Laabei et al. (2014)
Agar plate X-GAL test	✓					Nielsen et al. (2010)
**Cell culture and ex vivo techniques**
Bacterial survival with neutrophils	✓			✓		Schwartz et al. (2009); Pang et al. (2010), White et al. (2014)
Human neutrophil lysis test	✓			✓	✓	Nielsen et al. (2014)
Human keratinocyte cell lysis test	✓			✓	✓	Cech et al. (2012)
Rabbit erythrocyte lysis test	✓			✓	✓	Pang et al. (2010)
Virulence expression on reconstituted human epithelia	✓			✓		Paniagua-Contreras et al. (2014)
**Chromatography tools**
HPLC quantification of δ-hemolysin	✓	✓		✓	✓	Otto and Gotz (2000); Quave et al. (2011)
LC-MS detection of δ-hemolysin	✓			✓		Somerville et al. (2003)
MALDI-TOF/TOF MS detection of δ-hemolysin	✓			✓		Gagnaire et al. (2012)
HPLC quantification of PSM-α	✓	✓		✓		Chua et al. (2014)
IMS and MALDI-MS/MS characterization of metabolic products and PSMs	✓	✓		✓		Gonzalez et al. (2012)
Quantification of extracellular AIPs by UHPLC/MS	✓	✓		✓		Junio et al. (2013); Roux et al. (2014)
**Molecular techniques**
Transcriptional analyses by Northern blotting	✓			✓	✓	Gagnaire et al. (2012); Nielsen et al. (2014)
qRT-PCR	✓			✓		Park et al. (2007)
*agr::*P3 driven GFP reporter assay		✓	✓	✓	✓	Murray et al. (2014); Sully et al. (2014)
*agr*::P3 driven *lux* reporter assay		✓	✓	✓	✓	Subrt et al. (2011); Figueroa et al. (2014)
P3-*blaZ* reporter		✓		✓	✓	Nielsen et al. (2014)
AIP fluorescent reporter competition assay		✓		✓		Kavanaugh et al. (2007); Malone et al. (2007)
Vesicle lysis test		✓		✓		Laabei et al. (2014)

*^a^Testing throughput: Assay is amenable to the following throughput testing. L, low throughput testing, amenable to a small number of samples. M, medium throughput screening, amenable to 96-well plate assays and not requiring robotic liquid handling systems. H, high throughput screening, amenable to large samples, such as 96-well or 384-well microtiter plates with the assistance of robotics*.

*^b^EE: Requires the use of expensive equipment or core services, such as microplate readers, HPLC, mass spectrometer, and etc*.

*^c^SM: Adapted for testing small molecule inhibitors of the *agr* system*.

*^d^References: Select references are included and this does not represent the full body of literature for each assay*.

• Low throughput refers to techniques that require heavy manual personnel involvement, with little to no robotics. These are best suited to small-scale investigation of no more than a few drug candidates at a time and are most appropriate for the secondary or tertiary QSI lead validation stage in the discovery process.
• Medium throughput refers to those techniques which are amenable to screening hundreds of compounds. These methods are often conducted in micro-volume assays (e.g., with 96-well plates), require personnel for sample setup and processing, and incorporate some degree of automation (e.g., microtiter plate readers, autosamplers, etc.).
• High throughput refers to the heavy integration of automation into the experimental and computational approaches employed. High throughput screening (HTS) efforts can be used to rapidly screen large numbers (1000s to 100s of 1000s) of test compounds at rates that are impossible to duplicate with low- and medium- throughput approaches. Personnel involvement in liquid handling is more limited, and robotics systems are employed to reduce manual labor. Prior to implementation of HTS, it is recommended to use smaller pilot screens to calculate the Z-factor (also known as Z-prime or Z′) to determine the suitability of an assay to HTS applications (Zhang et al., 1999).

### AGAR PLATE ASSAYS

#### Blood agar plate assays

A simple method for assessing *agr* activity is to test the hemolytic activity of δ-hemolysin by streaking culture on a blood agar plate in combination with an intersecting streak of a β-hemolytic positive strain in the CAMP assay (Elek and Levy, 1950). While this inexpensive test can be used to detect *agr* activity, it has not yet been adapted for small molecule screening efforts.

Another useful assay for detecting hemolytic activity of *agr* translational products involves streaking strains on a TSA plate with 5% sheep blood, starting at the center of the blood agar plate which contains a β-hemolysin disk and moving outward (Schweizer et al., 2011). δ-hemolysin is detected through visualization of a zone of clearance created by synergistic activity with β-hemolysin near the center of the plate. While not very amenable to screening many QSIs (only one candidate at a time would be incorporated into the media of the plate), this could be easily adapted for examination of a QSI candidate against up to six strains at a time or as a means of secondary confirmation of QSI activity following HTS. Expected results would include zones of clearance along the streak of the agar control plate, and lack of zone formation along the growth streaks found on the agar plate containing the QSI candidate. Thus, it could be a simple useful test for assessing quenching activity against the four *agr* types on a single agar plate.

#### Phospholipase plate assay

**FIGURE 2:**
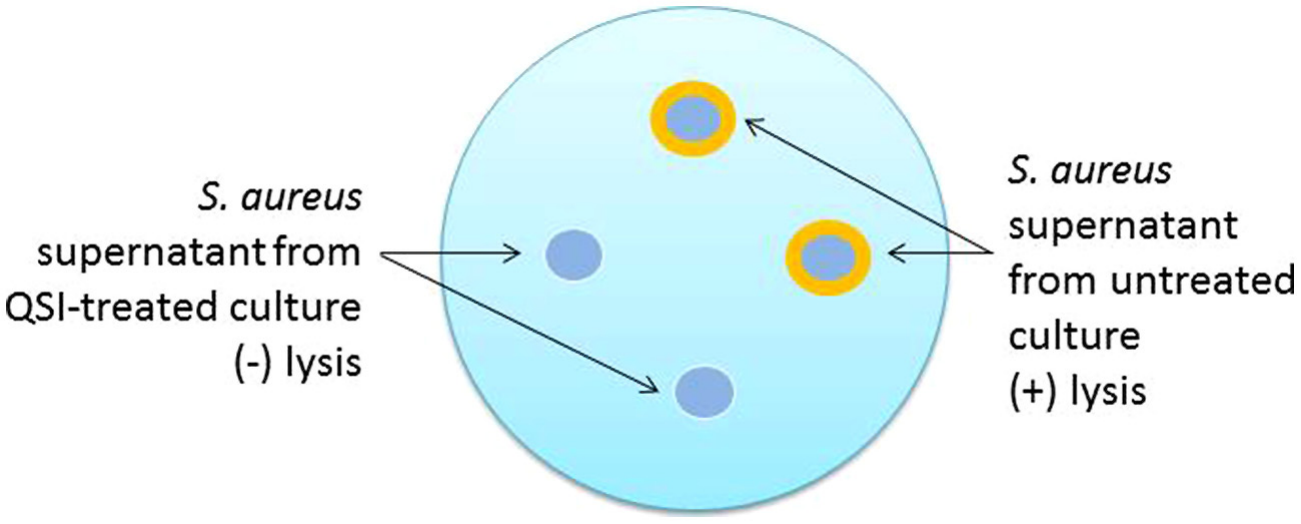
**Schematic representation of the phospholipase plate assay for the identification of quorum sensing inhibitor (QSI) activity.** In this test, 18 h cultures are grown either in the presence or absence of QSI, the supernatants harvested and sterile filtered, and then added to the punch holes made in the phospholipid plate. Following an additional 18 h incubation period, the plates are exposed to UV irradiation and photographed. The orange halos indicate the lytic activity of untreated supernatants. Supernatants from QSI-treated cultures would exhibit limited to no lytic effects and no orange halo.

In addition to the production of toxins, *S. aureus* also produces a number of enzymes, some of which exhibit lipolytic activity as (phospho)lipases (Laabei et al., 2014). A new version of the lipase plate assay involves a modification of the original method (Kouker and Jaeger, 1987) that incorporates olive oil (1%) and rhodamine B (0.001%) into the substrate added to the agar medium (Laabei et al., 2014). Holes are then punched into the agar and supernatant is added, the plate incubated, irradiated with UV light, and images captured for analysis. An orange halo emerging around the supernatant indicates the presence of lipase (**Figure 2**). Treatment of *S. aureus* with a successful QSI will not yield the halo zone in the agar dish, indicating the lack of lipolytic enzymes present in the supernatant.

#### Agar plate X-GAL test

**FIGURE 3:**
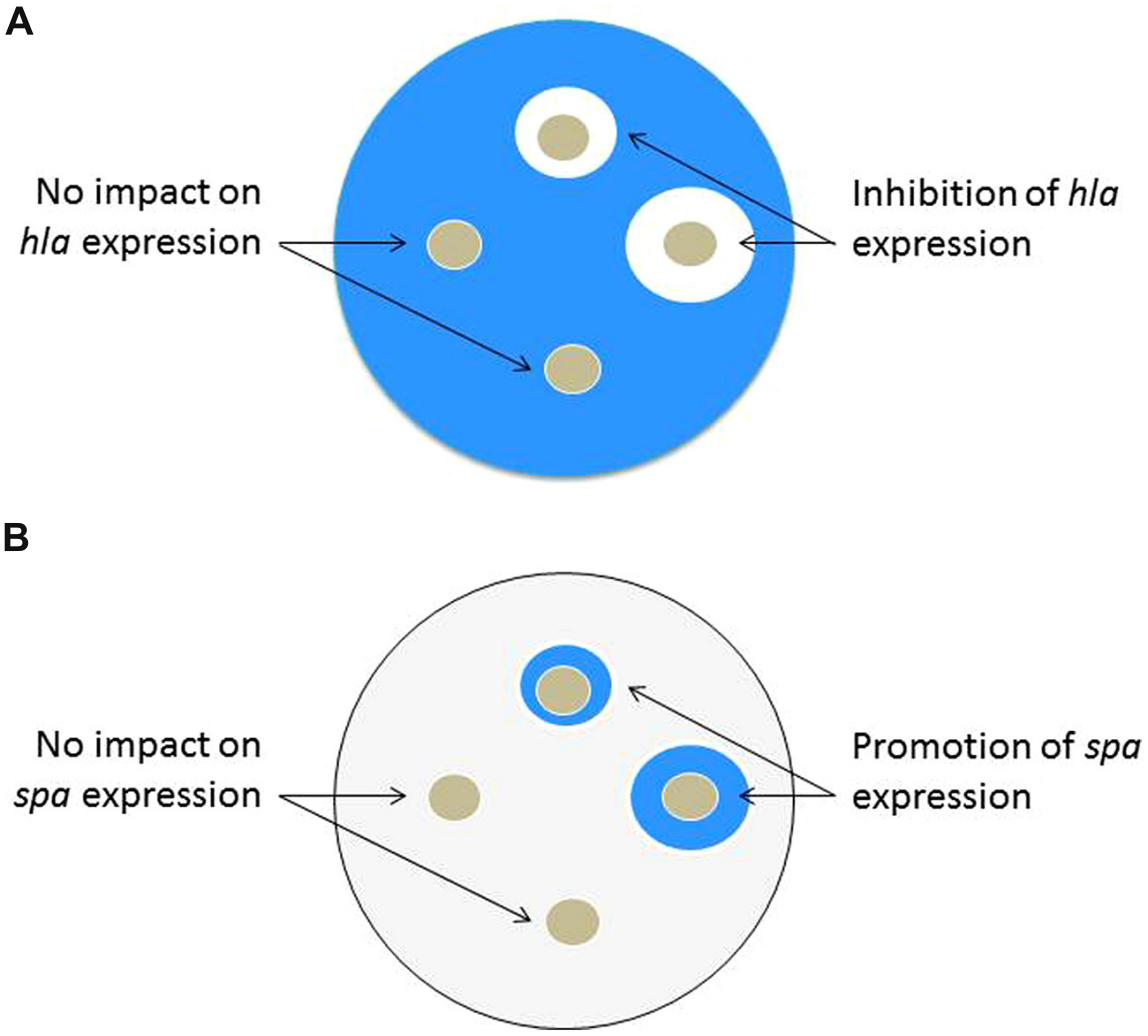
**Schematic representation of the X-GAL agar plate test, which can be used in QSI screening efforts of either individual compounds or more complex natural product extracts.** QSIs are identified by presence of both a white ring in the *hla::lacZ* plate and blue ring on the *spa::lacZ* plate. **(A)** *hla::lacZ* plate: following incubation of the plate containing the *hla* reporter, a white clearing surrounding the hole punch containing the QSI candidate indicates reduced expression of *hla* (α-toxin). **(B)** *spa::lacZ* plate: following incubation of the plate containing the *spa* reporter, a blue zone surrounding the hole punch containing the QSI candidate indicates increased expression of *spa* (Protein A).

*Staphylococcus aureus* strains with integrated *hla::lacZ* and *spa::lacZ* reporters have been used to screen for compounds with *agr* QSI properties (Nielsen et al., 2010). The *hla* gene encodes for α-toxin and the *spa* gene encodes for Protein A. As a chromosomal reporter, the *lacZ* gene encodes for the enzyme β-galactosidase that is readily detectable through colorimetric assays. Tryptic soy agar plates are made containing a diluted overnight culture of a reporter strain, the appropriate antibiotic to maintain the reporter, and X-GAL (5-bromo-4-chloro-3-indolyl-β-D-galactopyranoside) as a reagent to detect β-galactosidase activity. Following incubation, the plates containing *hla::lacZ* will become intensely blue due to high *hla* expression and thus production of β-galactosidase enzyme, while plates with *spa::lacZ* will become very light blue. Positive hits can be visualized by blue/white screening of the rings surrounding the hole punches containing putative QSIs. The intent of this approach is that QSIs added to the plate will prevent expression of *hla*, leading to white rings on the X-GAL plates. Simultaneously, a QSI will induce Protein A expression, leading to a blue ring with the *spa::lacZ* reporter **Figure 3**. Using the two reporters in parallel makes the assay more robust and has led to the successful identification of QSIs. A major advantage of this assay is its simplicity and low expense for screening both individual compounds and more complex natural product extracts. However, the *hla* reporter is also an indicator of SaeR/S regulatory expression and its interpretation must be considered carefully; see more on this in the section on qRT-PCR.

### CELL CULTURE AND *EX VIVO* TECHNIQUES

#### Bacterial survival with neutrophils

In this technique, the viability of *S. aureus* expressing superfolder green fluorescent protein (sGFP) in the presence of human neutrophils is assessed by flow cytometry. Following phagocytosis, fluorescence of *S. aureus* containing neutrophils is measured as a marker for cell viability (Schwartz et al., 2009; Pang et al., 2010; White et al., 2014). If the QSI candidate successfully blocks *agr* system function, then the impacted *S. aureus* cells will not be able to release toxins to damage the neutrophils. In turn, the *S. aureus* cells would be left susceptible to neutrophil attack resulting in a loss of sGFP signal. Toxins under *agr* regulatory control have been linked to intracellular survival phenotypes (Wang et al., 2007). The challenge is that this assay is not particularly well suited for screening studies of a large chemical library. It is not known whether an exogenously added compound could impact the *agr* regulatory activity following phagocytosis. However, if the technical details on the experiment could be developed, it might offer insight into the efficacy of specific compounds in blocking *agr-*regulated virulence pathways responsible for neutrophil lysis.

#### Cytotoxicity testing with *S. aureus* supernatants

Supernatants harvested from *S. aureus* cultures grown in the presence (or absence) of QSI candidates can be examined for the presence of secreted virulence factors that cause serious damage, and even lysis, of human cells. The following two assays make use of the lactate dehydrogenase release (LDH) cytotoxicity assay to measure the impact of sterile-filtered supernatants on the structural integrity of human cell lines. In addition to LDH tests, useful image data can also be collected of the cells following exposure to treated and untreated supernatants with the aid of fluorescent stains (e.g., LIVE/DEAD or DAPI) and fluorescent microscopy.

***Human neutrophil lysis test***. This assay examines the ability of QSIs to quench the *agr* pathway to a degree that prevents production of virulence factors responsible for neutrophil lysis. PSMs have been identified as the main lytic agents in this process (DeLeo et al., 2009; Pang et al., 2010; Surewaard et al., 2013; Cheung et al., 2014). Briefly, cultures of *agr-*active strains are grown in the presence (and absence) of the QSI under investigation. Human neutrophils are then exposed to the sterile filtered supernatants, and lysis is examined using the LDH cytotoxicity test (Nielsen et al., 2014). If the QSI candidate is, indeed, blocking production of virulence factors, the LDH output for neutrophils exposed to the QSI-treated supernatant will not show statistically significant difference from the spontaneous control neutrophils (which have not been exposed to any supernatant, but just sterile growth media).

***Human keratinocyte cell lysis test***. This assay examines the impact of QSI candidates on toxin production and viability of human keratinocyte (HaCat) cells. Here, *S. aureus* is grown in the presence of the QSI candidate or vehicle control, and the supernatant is harvested and sterile filtered prior to exposing to HaCat cells. Similar to the human neutrophil lysis test, this assay uses an LDH kit to detect cell lysis following exposure to the toxin-containing supernatant (Cech et al., 2012). Successful QSI candidates will exhibit no HaCat lysis above that exhibited by the media control.

#### Rabbit blood cell lysis test

Rabbit erythrocytes prepared from defibrinated blood are exposed to the supernatants of *S. aureus* treated with the QSI candidate or vehicle control. Following a period of incubation at room temperature, the degree of lysis is quantified by loss of turbidity [as measured by a plate reader at an optical density (OD) of 630 nm]. This test is a useful measure of *agr* function as it evaluates the presence of hemolysins in the supernatant, representing downstream translational products of RNAIII (Pang et al., 2010). The presence of hemolysins in the supernatant is confirmed by loss of red blood cell turbidity, and thus successful QSI candidates will demonstrate no change in turbidity from the media control. Alternatively, the change in release of hemoglobin from the lysed red blood cells can be monitored.

#### Virulence expression on reconstituted human epithelia

A new *in vitro* infection model uses reconstituted human epithelium (RHE) for assessing the expression of virulence genes. Here, *S. aureus* is grown on the RHE surface, and following a period of incubation, bacteria are harvested for RNA purification, reverse transcription, and subjected to real-time polymerase chain reaction (RT-PCR) using primers (e.g., *spa, cna,* and *agr*) for virulence gene profiling (Paniagua-Contreras et al., 2014). Although originally developed as a method to compare the profiles of a collection of clinical isolates, this method could provide interesting insight into the activity and mechanism of action of QSI lead compounds with regards to *agr* mediated virulence in an infection model.

### CHROMATOGRAPHIC TOOLS

#### Quantification of δ-hemolysin

**FIGURE 4:**
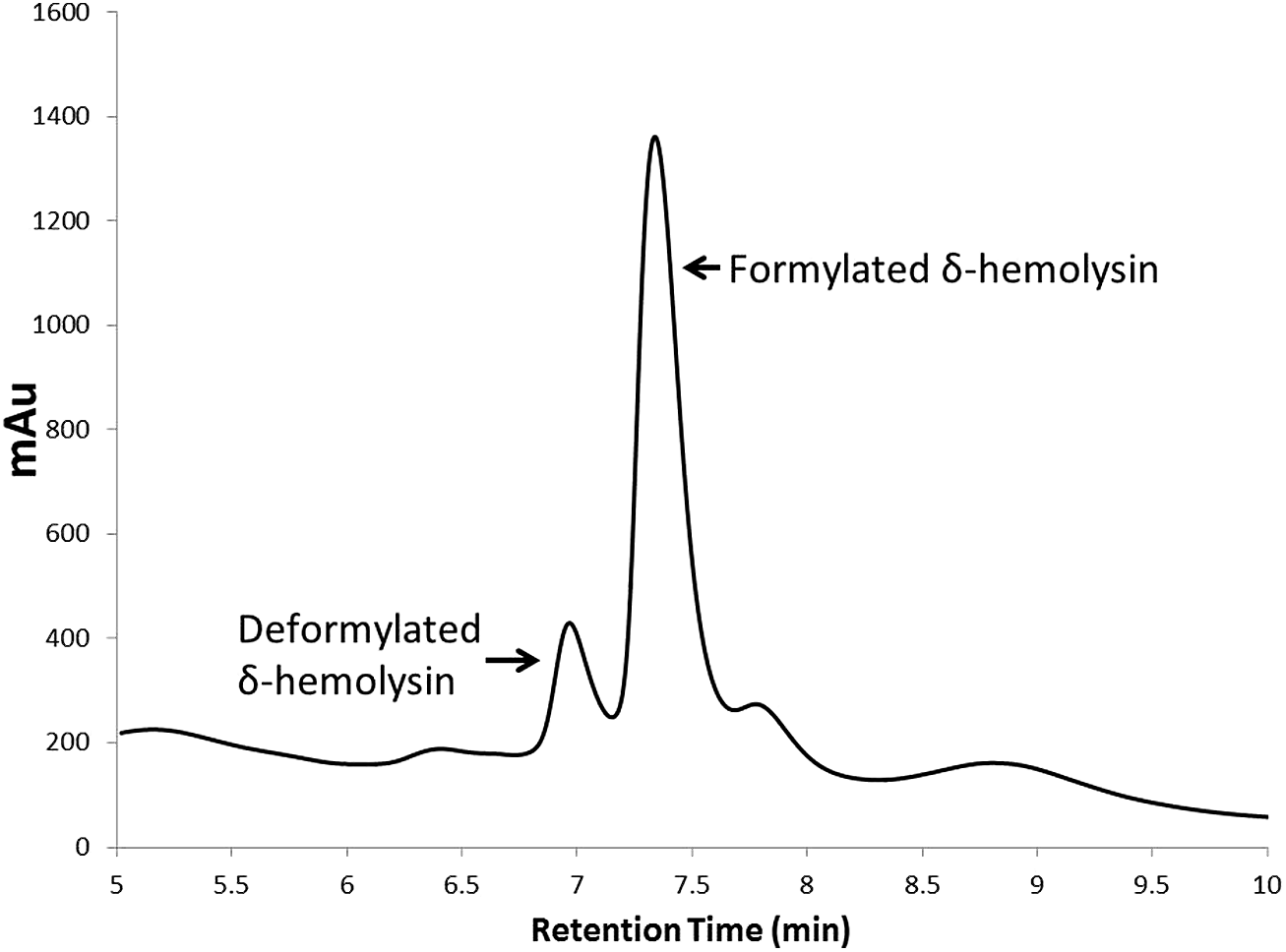
**High performance liquid chromatography chromatogram of deformylated and formylated δ-hemolysin present in a USA500 (NRS385) *S. aureus* culture supernatant.** Quantification of either the total peak height or peak area allows for accurate quantification of δ-hemolysin production in cultures grown in the presence (or absence) of QSIs. This technique is also very useful in dose-dependent activity studies on QSIs.

Staphylococcal toxins represent downstream products of RNAIII, and their detection and quantification is a useful marker for assessing *agr* activity. High performance liquid chromatography (HPLC) has emerged a useful tool in medium-throughput small molecule activity screening efforts. For example, a method for detecting δ-hemolysin in supernatant by RP-HPLC (Otto and Gotz, 2000) was modified to screen a library of medicinal plant extracts for QSI natural products (Quave et al., 2011). The advantages of this system over Western blots for detection of δ-hemolysin in drug screening efforts is the medium-throughput nature of the test (if the HPLC is equipped with an autosampler), highly reproducible quantification of the individual formylated and deformylated δ-hemolysin peaks, and limited sample preparation requirements as the supernatant can be injected directly onto the HPLC column. This technique is especially useful for determination of dose-response trends of a QSI, in which either the sum of total peak area or total peak height of the formulated and deformylated δ-hemolysin can be measured and plotted (**Figure 4**).

In addition to HPLC, mass spectrometry (MS) methods, including liquid chromatography MS (LC-MS; Somerville et al., 2003) and Whole Cell Matrix Assisted Laser Desorption Ionization Time-of-Flight/Time-of-Flight MS (MALDI-TOF/TOF MS; Gagnaire et al., 2012) have also been successfully used to detect δ-hemolysin. These MS methods could prove very useful if adapted for medium-throughput QSI screening efforts.

#### Quantification of PSM-α

As discussed above, *agr-*regulated PSMs are very effective at recruiting, activating and lysing human neutrophils, and as such, they are key to the pathogenesis of *S. aureus* infections (Otto, 2010). Similar to the detection and quantification of δ-hemolysin, an RP-HPLC method has also been used to detect the deformylated and formylated forms of PSM-α in culture supernatants (Chua et al., 2014). Moreover, new imaging MS (IMS) methods have been developed to characterize the microbial metabolic output of strains (including PSMs), which can also be further characterized by MALDI-MS/MS (Gonzalez et al., 2012).

#### Quantification of extracellular AIPs

Among known *S. aureus* strains, there are four different classes of *agr* systems, each recognizing a unique AIP structure (referred to as *agr*-I, *agr*-II, *agr*-III, and *agr*-IV, and correspondingly their cognate signals are termed AIP-I through AIP-IV). Interestingly, these different classes of AIPs exhibit cross-inhibition to one another, and some studies have focused on screening synthetic AIP mimetics (Tal-Gan et al., 2013). Quantitative methods have recently emerged for analyzing AIP-I (Junio et al., 2013) and AIP-III (Roux et al., 2014) using ultrahigh performance liquid chromatography (UHPLC) coupled to electrospray ionization MS with an LTQ Orbitrap mass spectrometer. This technique is particularly useful in monitoring time-dependent release of AIPs and could be adapted for assessment of QSI candidate impact on AIP release to the extracellular environment.

### MOLECULAR TOOLS FOR IDENTIFYING QSI

#### Transcript analysis

One of the original approaches to assessing *S. aureus* quorum sensing is through direct analysis of transcripts. Northern analysis is the classical approach and the high expression level of *rnaIII* transcript and its stability make it a useful candidate for tracking. More recently, quantitative reverse transcriptase PCR (qRT-PCR) has become a standard alternative approach used by many laboratories.

***Transcriptional analyses by Northern blotting***. Probes directed at RNAIII transcript can be used to assess *agr* activity (Kjaerulff et al., 2013). For example, in a study by Nielsen et al. (2014), strains were grown in the presence of a QSI lead molecule, RNA was purified, and Northern blot analysis on *agrA* and *psmα* was performed. Likewise, in another study on QSI candidates, CA-MRSA isolates were grown in the presence of solonamides and Northerns were performed on *rnaIII*, *spa*, and *hla* transcripts to assess differences in virulence gene expression between the treated samples and vehicle control (Mansson et al., 2011). While this technique is not amenable to large compound screens, it can provide useful information on the impact of a lead QSI candidate on virulence gene transcription.

***qRT-PCR***. Quantitative reverse transcriptase PCR (qRT-PCR) with primers for *agr* can be used to monitor *agr* function (Park et al., 2007). The best targets for assessment are *rnaIII*, *psmα*, and *psmβ* transcripts because these are under direct AgrA control (Queck et al., 2008). As indicated above, Protein A (*spa*) is also informative due to its opposite response when *agr* is inhibited. Although the *hla* transcript, encoding α-toxin, is a popular choice for an assessment of *agr* function, its interpretation is more difficult. The predominant action of the *agr* system is to control protein translation of Hla. In other words, an *agr* mutant does not produce Hla protein, but this mutant will show only minimal changes (typically twofold) to *hla* transcript levels. Thus, small molecule inhibitors that greatly reduce *hla* transcription should be viewed with caution since the dominant transcriptional regulator of this gene is the SaeR/S system (Flack et al., 2014).

#### Reporter tools

As an alternative to direct analysis of transcripts, transcriptional reporters for *S. aureus* quorum sensing dependent promoters are often used for monitoring *agr* system function and identifying QSI. The *agr* P3 promoter is most commonly used for this purpose due its strong output and large dynamic range. In this section, different reporter-based approaches are outlined using both fluorescent and luminescent markers, and these approaches can be applied for monitoring *agr* system function or identifying a QSI.

***Fluorescent reporters***. A number of fluorescent reporter strains have been developed for use in studies aimed at understanding the mechanistic underpinnings of the system and for small molecule QSI screening efforts (Malone et al., 2009; Shojima and Nakayama, 2014). The most commonly used ones contain either a GFP, or a similar emitting yellow fluorescent protein (YFP), that is linked to the *agr* P3 promoter. Although the *agr* P2 promoter has been utilized, it has much weaker output and a more limited dynamic range. When these reporters are used in conjunction with a microplate reader that has the capacity for reading OD for growth and fluorescence, these strains allow for the detection of either promotion or inhibition of the *agr* system. Moreover, these tools are readily adaptable for high-throughput screening of small molecule repositories. For example, a screen of a 24,087 compound library using a reporter strain with *agr::*P3 driven GFP expression resulted in the discovery of savirin as a small molecule inhibitor of AgrA, which was further supported by additional *in vitro* and *in vivo* studies (Sully et al., 2014). Successful QSI candidates exhibit growth ODs similar to those of the vehicle control, but with significantly reduced fluorescence outputs, signaling loss of *agr* P3 promoter activation.

***BlaZ reporter***. The *agr* P3-*blaZ* reporter has been used in some of the original efforts to identify *S. aureus* QSI (Mayville et al., 1999; Lyon et al., 2000). The *blaZ* gene encodes for β-lactamase, and the activity of BlaZ enzyme can be easily tracked using nitrocefin assays. The technique is also useful for mechanistic investigations of *agr* system function (Geisinger et al., 2012). Recently, the BlaZ reporter has been used in efforts to characterize the natural product solonamide B as an AgrC antagonist (Nielsen et al., 2014). The reporter could be used to identify *agr* inhibitors in the medium throughput screening models described above.

***AIP competition assay***. Autoinducing peptide competition can also be tracked through the use of fluorescent reporters (see section Fluorescent reporters) for the different *agr* and AIP types (Malone et al., 2007). Reporter strains are grown in the presence of the appropriate antibiotic to maintain the plasmid, and then once reaching an established OD, are aliquotted and mixed with either control media or competing AIP. Cell density and fluorescence is measured with a microplate reader to examine impact on growth and *agr* activity. A decline in fluorescence, without impacting growth, is interpreted as QSI activity. This approach was successfully used to test Type I signal peptidase inhibitors for impact on *agr* function (Kavanaugh et al., 2007), and it could be amenable to screening compound libraries.

***Vesicle lysis test***. A new method to detect small amphipathic α-helical peptides and measure *agr* activity has been developed (Laabei et al., 2014). The synthetic lipid vesicle type used in this assay was chosen specifically for its selectivity to PSMs and δ-hemolysin, excluding other known *S. aureus* toxins. The vesicles prepared for this test comprise a similar level of cholesterol as that found in erythrocytes (20–25%) and includes two very common membrane phosphoglycerides: phosphatidylcholine and phosphatidylethanolamine. Following preparation of the vesicles, bacteria can either be normalized to a specific OD for corresponding colony forming units per mL (CFUs/mL) or supernatants can be harvested for use in the experiment. The vesicle solution and bacteria (or supernatant) are then mixed and fluorescence is monitored with a fluorescent detector. An increase in normalized fluorescence over the untreated control (with no toxin) indicates an increase in toxin-mediated vesicle lysis. Some advantages of this assay include that it is relatively quick to perform and is amenable to 96-well plate medium throughput testing using a plate reader with fluorescent detection abilities. It is reported to have greater accuracy in correctly identifying strains with active *agr* systems than the CAMP assay (Laabei et al., 2014). It could be adapted to QSI screening efforts perhaps either by inclusion of QSI candidates in the test containing live, OD-standardized culture or by using supernatants of cultures grown in the presence of QSI candidates.

***Luminescent reporters***. Strains with *lux* reporters that link the *agr* system to a bioluminescence response have been developed (Park et al., 2007; Subrt et al., 2011; Figueroa et al., 2014). These constructs utilize the *Photorhabdus luminescens luxCDABCE* genes that self-produce light without the need for additives. Since the *lux* genes are often poorly expressed in Gram-positive bacteria such as *S. aureus*, improved constructs have been developed that increase production of the fatty acid aldehyde substrate needed (Mesak et al., 2009). The *agr* P3 promoter has been coupled to this improved construct for tracking quorum sensing function (Subrt et al., 2011). Recently, this construct has been moved to clinical isolates and used successfully to screen for QSIs (Figueroa et al., 2014). As described above for fluorescent reporter screens, in comparison with the vehicle control, successful QSI candidates yield little to no change to growth OD but significant reduction in luminescent output.

## CONCLUSION

At a time when the field of medicine stands on the precipice of the post-antibiotic era (CDC, 2013), the anti-virulence strategy may represent a viable solution to addressing the emerging therapeutic gaps presented by growing trends in antibiotic resistance (Spellberg et al., 2013). This strategy aims to interfere with cell–cell communication, or quorum sensing, through which single-celled organisms coordinate gene expression in adaptive measures to enhance survival in their respective environments, including their human hosts. Discovery of small molecule QSI candidates represents the first step in the path toward integration of an anti-virulence strategy into the treatment and management of *S. aureus* infections.

Over the past decade, the “toolbox” for studying the *S. aureus agr* QS system and searching for novel QSI candidates has expanded greatly. In addition to the standard, lower throughput Western and Northern blots, we now have a suite of fluorescent and luminescent reporter strains as well as various chromatographic techniques at our disposal for the study of this important virulence target. Nevertheless, most of these techniques have not yet been further developed or modified for applications in medium- to high-throughput screening efforts for the identification of novel QSIs. One caveat concerning studies focused on *agr* as a target is the potential impact of test candidates on growth. This is something that must be closely monitored as growth-dependent inhibition can yield a false-positive result for QSI activity, and thus QSI activity should be explored at sub-MIC concentrations. Likewise, QSIs inhibiting certain virulence factors (i.e., α-toxin) require additional evaluation, as this may not reflect true *agr* inhibition, but rather alternative (or additional) impact on the *S. aureus* SaeR/S system. Future work should build on these existing resources, optimizing them to further support efforts for the identification and mechanistic analysis of small molecule inhibitors of this highly clinically relevant target.

## AUTHOR CONTRIBUTIONS

Cassandra L. Quave and Alexander R. Horswill conceived of and wrote this review together.

## Conflict of Interest Statement

The authors declare that the research was conducted in the absence of any commercial or financial relationships that could be construed as a potential conflict of interest.
